# Whole genome sequence analysis of CPV-2 isolates from 1998 to 2020

**DOI:** 10.1186/s12985-023-02102-2

**Published:** 2023-07-03

**Authors:** Sajed Sarabandi, Hadi Pourtaghi

**Affiliations:** 1 Department of Pathobiology, Islamic Azad University, Karaj Branch, Karaj, Iran; 2 Department of Microbiology, Islamic Azad University, Karaj Branch, Karaj, Iran

**Keywords:** CPV-2, N93K, A5G, VP2, Sequence analysis

## Abstract

Canine parvovirus-2 (CPV-2) is a virus with worldwide spread causing canine gastroenteritis. New strains of this virus have unique characteristics and are resistant to some vaccine strains. Therefore, understanding the root causes of resistance has proven to be of increasing concern to many scientists. This study collected 126 whole genome sequences of CPV-2 subtypes with specific collection dates from the NCBI data bank. The whole genome sequences of CPV-2 collected from different countries were analyzed to detect the new substitutions and update these mutations. The result indicated 12, 7, and 10 mutations in NS1, VP1, and VP2, in that respective order. Moreover, the A5G and Q370R mutations of VP2 are the most common changes in the recent isolates of the CPV-2C subtype, and the new N93K residue of VP2 is speculated to be the cause of vaccine failure. To summarize, the observed mutations, which are increasing over time, causes several changes in viral characteristic. A comprehensive understanding of these mutations can lead us to control potential future epidemics associated with this virus more efficiently.

**Supplementary Information:**

The online version contains supplementary material available at 10.1186/s12985-023-02102-2.

## Introduction

Canine Parvovirus 2 (CPV-2) is a member of the Parvoviridae family, which typically causes gastroenteritis and myocarditis in wild and domestic canines [1]. According to recent studies [2], three antigenic variants of CPV-2 have been classified based on the amino acid 426 of capsid protein VP2, including CPV-2a (asparagine N426), CPV-2b (aspartic acid D426), and CPV-2c (glutamic acid E426). The entire genome of CPV-2 consists of near 5Kbp nucleotides, which contain two open reading frames [3], the first one encoding non-structural proteins NS1 and NS2, with the second one encoding capsid proteins VP1 and VP2 [4]. The capsid encapsulates the viral genome, transports it to the host cell, where it is subsequently released. As a result, any change in the capsid structure may increase viral proliferation and virulence. Several studies have been conducted recently to compare and demonstrate the mutations among these variants [5, 6]. The result of these studies highlighted various alterations in the amino acid sequences that might result in novel behavior. Some researchers have also pointed out that these mutations may cause vaccine resistance [7]. In a study by Zhou [4], 1679 sequences of CPV-2 from 1978 to 2015 were examined, and the result identified three single mutations (F267Y, Y324I, and T440A), which may be responsible for vaccine failure. Due to the importance of these alterations, this study investigates the CPV-2 genome sequence from 1998 to 2020 to distinguish any new changes, highlight notable mutations, and examine the evaluation process of CPV-2. This may help more thoroughly predict future viral behavior, such as in the case of vaccine failure.

## Materials and methods

With the goal of detecting mutations in CPV-2, 126 complete genome samples were observed in the NCBI data bank (All the information along with accession number can be found in Additional file 1). The FASTA sequences of samples were downloaded with a specific collection date and country name. In this study, samples collected after 2015 are referred to as newer samples in a relative manner. All the samples were sorted based on their collection date and aligned with each other. Based on the amino acid 426, samples were divided into three distinct sub-types, including CPV-2a (35), CPV-2b (26), and CPV-2c (65). The data with FASTA format was imported to the MEGA-X for the alignment analysis. At this point, the genome sequences are translated into the protein sequence to facilitate future analysis. In the next step, data is imported to the R program to extract any mutation from protein sequences. The amino acid mutations observed only in one sample were ignored to increase the accuracy. Due to the role of VP2 in viral pathogenicity, the evolution of amino acid mutations in this protein was illustrated in two graphs designed using the python program. Finally, the 3D structure of VP2 was downloaded from SWISS-MODEL (https://swissmodel.expasy.org), and four crucial amino acid mutations with a high range of changes were highlighted in the 3D model of the protein with the Chimera program.

## Result

Based on the bioinformatic analysis, a total number of 121 amino acid substitutions were discerned among all the samples, of which 67 were related to NS1 protein, 27 to VP1, and 27 to VP2.

### NS1

NS1 significantly influences the pathogenicity and cytotoxicity of canine parvovirus 2 [8]. Table 1 lists the amino acid mutations found in NS1 sequences. One of the altered amino acids, M11K, was only spotted in the Australian isolates of the CPV-2c subtype. In samples obtained after 2015, a little more than 60% of CPV-2c (15 of 24) and one sample of each CPV-2a and CPV-2b had the amino acid alteration I60V. Moreover, most of the N351K (28 of 31) and N361S (5 of 6) mutations were observed in the South American countries’, including Argentina, Brazil, Paraguay, Uruguay, and Peru. The Y544F and E545V are two amino acid mutations that were witnessed in each of the CPV-2a, CPV-2b, and CPV-2c subtypes, specifically in the CPV-2c samples that were collected after 2015. In contrast, E572K mutations did not occur in CPV-2c but were observed in 20 CPV-2a and CPV-2b subtypes isolates. Finally, the amino acids 630 was significantly (16 of 24) altered in the recent years’ samples of CPV-2c.

**Table 1:** NS1 mutations

Amino acid mutation	Amino acid location	Number of mutated samples*
M11K	11	4
K19R	19	6
I60V	60	15
N351K	351	31
N361S	361	6
Y544F	544	49
E545V	545	23
E572K	572	20
E583K	583	18
L597P	597	17
L630P	630	17
D668E	668	9

Mutation types, location, and number of amino acid mutations in NS1

*The total number of sample was 126

### VP1

Most of the viral capsid consists of VP2 amino acid sequences and only a few are related to VP1. VP1 contains the whole sequence of VP2 with an extra, unique N-terminal sequence [9]. The N-terminal sequence of VP1 is associated with phospholipase A2 which helps the virus release from the endosome [10]. Due to the role of N-terminal in VP1, any changes in the amino acid sequence of this region is presumed to affect virus replication and virulence. Our bioinformatic analysis indicates several nucleotide mutations in the VP1. Most of the observed mutations are in bps 2308 to 2380 nucleotides of the genome, which do not participate in encoding any amino acids in VP1; however, some of the mutations result in amino acid sequence alterations. The study indicates that all five Vietnamese CPV-2c samples have the T112I and L125F mutations. Additionally, the L125F mutation was found in 3 of 15 recent samples of CPV-2a, with the A131T mutation found in 10 of 24 recent samples of CPV-2c (Table 2). These mutations may suggest an effect on virus replication and virulence leading to new behavior and characteristics.

**Table 2:** VP1 mutations

Amino acid mutation	Amino acid location	Number of mutated samples*
A93V	93	2
S104L	104	2
T109A	109	2
T112I	113	5
K116R	116	19
L125F	125	10
A131T	131	15

Mutation types, location, and number of amino acid mutations in VP1

*The total number of sample was 126

### VP2

The VP2 consists of 584 amino acids. This protein has at least four loops and plays a significant role in viral pathogenicity [11]. A total of 27 mutations were observed in the set of analyzed VP2 amino acid sequences. Several studies reported the importance of new mutations, including Y267F, Y324I, and T440A [12, 13]. Our result indicates that amino acid 5 had G5A mutations, specifically in 14 of 15 recent CPV-2c samples. Two new mutations, V300G and T389N, were observed in the samples from Iran and Japan. The amino acid 324, which recently changed from the original Y to I, had a novel Y324L mutation in recent samples of CPV-2b from Brazil. Moreover, a Q370R mutation has been observed in the recent samples of CPV-2c. We also noted that the amino acid 447 had been altered from I to M only in the recent samples of CPV-2c in Vietnam. These mutations could potentially lead to new behavior and affect viral pathogenicity. More research is required to examine the role of the previously mentioned mutations. Due to the importance of VP2 in pathogenicity and the high mutation rate in amino acids 5 and 370, the evolution process of these amino acids has been subject to investigation below (Table 3).

**Table 3:** VP2 mutations

Amino acid mutation	Amino acid location	Number of mutated samples*
A5G	5	15
N93K	93	2
F267Y	267	37
G300V	300	2
Y324L	324	7
Q370R	370	14
T389N	389	3
T440A	440	89
S440A	440	12
I447M	447	3

Mutation types, location, and number of amino acid mutations in VP2

*The total number of sample was 126

### The evolutionary process of amino acid 5 and 370 in VP2

**Fig. 1:**
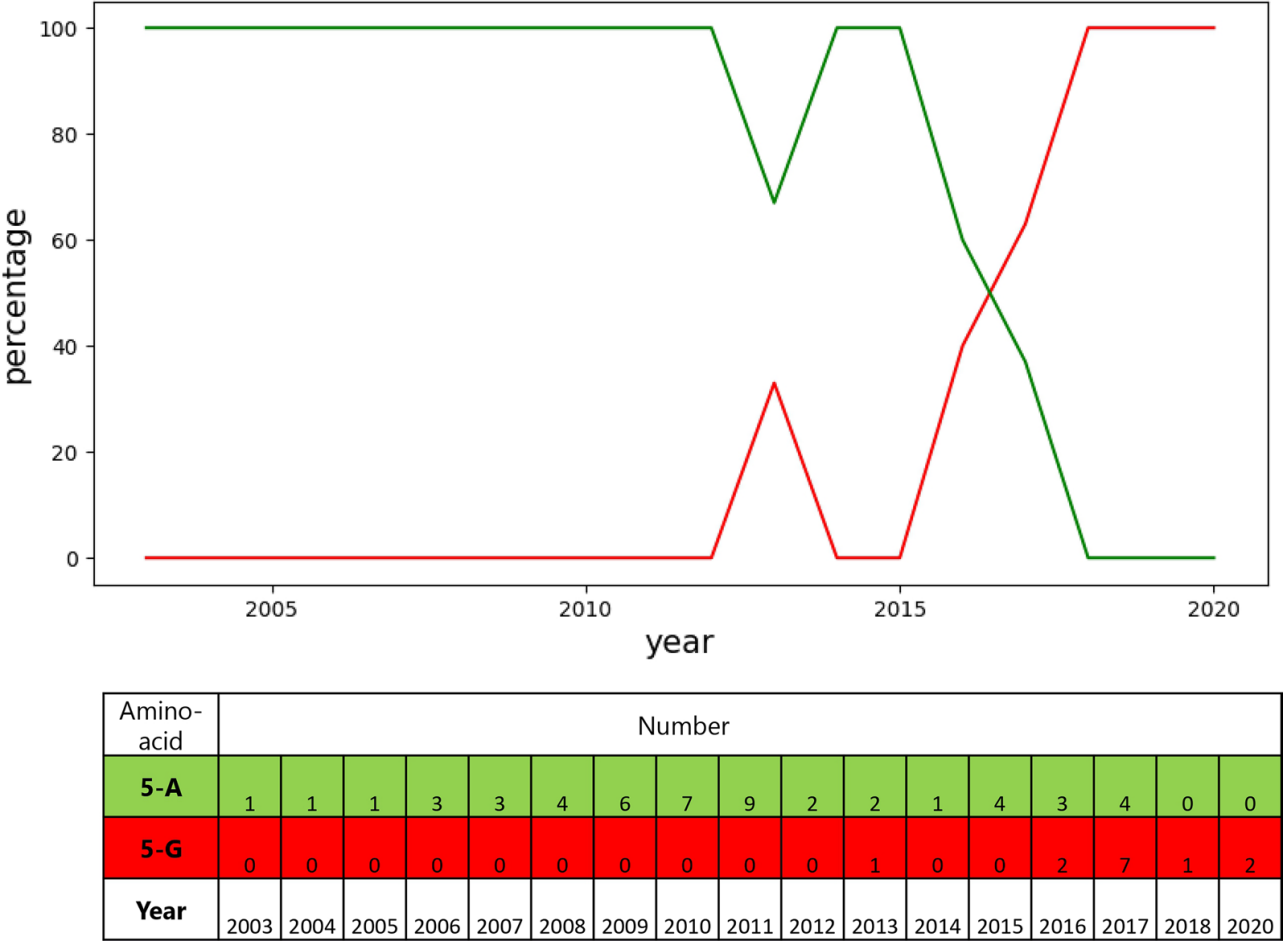
The amino acid percentages and numbers at the location 5 of VP2

**Fig. 2:**
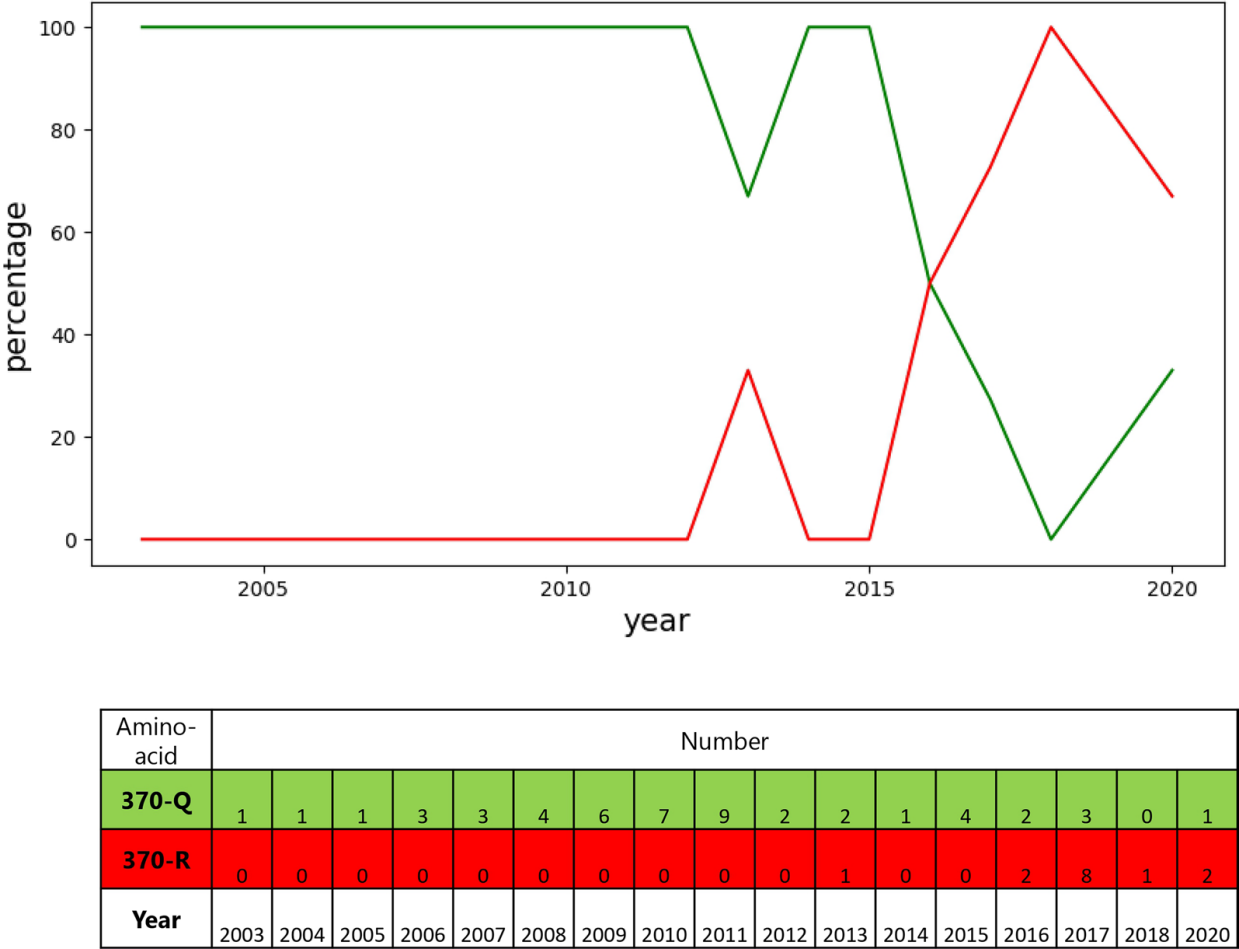
The amino acid percentages and numbers at the location 370 of VP2

Among all of these mutations, several amino acid substitutions increased over time. The viruses with these substitutions are undergoing an evolution that may help them replicate more than other variants. Zhou et al. [4] mentioned the evolution of amino acids 267, 324, and 440. It was proposed that the mutation of amino acid 440 might cause viral antigenic drift and lead to vaccine failure. This study distinguishes two more amino acid substitutions in the CPV-2c subtype (G5A and R370Q), which increased over time (Figs. 1 and 2). According to the sequence samples covered in this study, the first observed mutations of amino acids 5 and 370 were in 2013 in Vietnam isolates. Figures 1 and 2 show that these mutations are growing in frequency over time. Despite the fact that these amino acids are not located in any protein loop, it has been speculated that the amino acid 370 enhance viral pathogenicity, which will be discussed in the following paragraphs.

### VP2 mutations on the 3D model

**Fig. 3:**
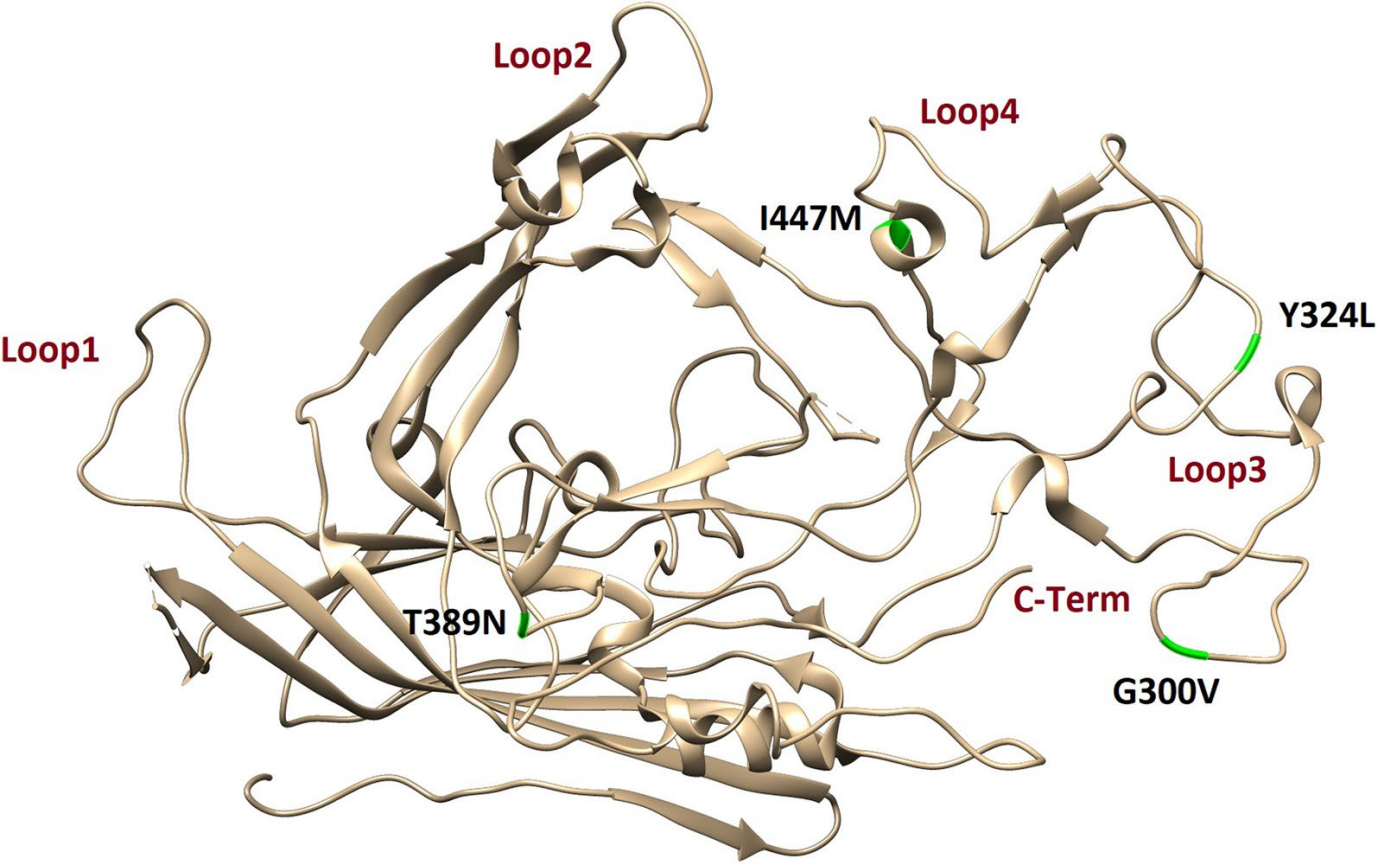
The 3D structures of VP2 and the location of the important mutations

A protein loop is consisted of two irregularly connected secondary structures [14]. Surface protein loops play a crucial role by binding to various biomolecules [15]. Due to the importance of VP2 in viral pathogenicity, the 3D model of VP2 has been retrieved from the Swiss-model server, and significant amino acid mutations have been highlighted in this model. The VP2 protein consists of at least four loops. Any modifications to the amino acids near these loops may result in new characteristics. As mentioned, amino acids 5 and 370 are not near any loop and Zhou et al. 2017 already highlighted the position of amino acids 267, 324, and 440 [4]. Therefore, in this study, the position of amino acids 300, 324, 389, and 447 have been marked in the VP2 3D structure (Fig. 3). The result indicates that the amino acids 300 and 389 are located in loops 3 and 5, respectively; thus, their mutations may alter viral behaviour. Recent studies demonstrate that the change in amino acid 300 determines the host range of CPV-2 [16]. However, the latter amino acid was not located near any loops. As a result, they might not cause any important alterations in viral characteristics.

## Discussion

The CPV-2 is a DNA virus with 10-4 mutation sites per year [17] that is presumed to change viral characteristic. Therefore, this study aims to provide explanation on viral behavior by attempting to understand some these substitutions. NS1 protein plays a crucial role in viral pathogenicity and cytotoxicity [8, 18]. The mutations of N351K and N361S in NS1 were observed in all the South American samples within the analyzed set. The characteristic of these mutations is yet to be examined. The mutations of I60V and L630P in NS1 previously reported by Hualei Wang et al. [13] have been observed in recent isolates from numerus countries indicating an increase in the frequency of these substitutions among all examined CPV-2 variants. The Increase in the population of any specific mutation could hint at new beneficial characteristics such as pathogenicity, drug resistance, and viral replication, possibly causing vaccine failure [19]. These unique characteristics may help the virus increase the replication rate and host range. The same supposition applies as in the case with VP2 mutations assessed below, making them of potential value to research.

Illustrating the evolutionary process of VP2 reveals that G5A and Q370R mutations in CPV-2c have increased over time. The recent changes in the CPV-2 behavior, for instance vaccine failure, could be caused by the increase in commonality of these two amino acid mutations within the observed set. Amino acid 5 located in the N-terminal part of the VP2 could possibly be related to ligand binding, integration into the cell membrane, and receptor internalization [20]. However, the role of this amino acid is yet to be identified. As regarding Q370R mutation, It is believed to have two aspect functions [21] It may correlate with the ability of CPV-2 hemagglutination because it is near the amino acids 375 and 377. Also, this amino acid is near amino acids 379 and 384, which may affect the CPV-2 host range. In this study, no meaningful changes have been observed at the mentioned sites. As a result, the role of Q370R mutation could not be identified.

The CPV-2 viral capsid protein is coded by two DNA sequences, VP1 and VP2. The former, VP1, has a unique coding sequence for the protein’s N-terminal part, making it longer than the VP2. The N-terminal has several regions acting as nuclear localization signals (MAPPAKRARRGLV) [22]. This part helps the virus infect the cell and introduce its genome to the host cell, playing an active role in replication. As previously mentioned, most of the observed mutations in the VP1 sequences did not code any amino acids. Therefore, these mutations might not have an impact on viral replication. These mutations are in the beginning part of the sequences; thus, may have other functions, including regulating gene expression [23]. Any changes in this part did not affect the viral capsid protein’s chain. On the other hand, it has been speculated that other mutations, which are in the N-terminal site may alter the viral replication rate by changing the structure [9]. Understanding these mutations’ characteristics can help us find a way of targeting this region and controlling the viral replication rate.

VP2 is the main DNA sequence coding the capsid protein in CPV-2 and plays a crucial role in viral pathogenicity. Numerous studies reported the VP2 mutations and their features [24, 25]. Based on our observation, T440A is the most common substitution of VP2 located at the top portion of loop four. It has been expected that the change of threonine to alanine in this location causes antigenic drift, which leads to vaccine failure. N93K is also a reported substitution in Minute virus of mice, another member of the Protoparvovirus genus, with a genome similar to that of CPV-2. In a study by Agbandje et al. 1998 [26], it has been mentioned that this substitution is related to host range determinants, which change the antigenic region of VP2. In this study, we observed this mutation in a sample from Iran and Japan in 2020 and 2017, respectively. The change in amino acid 93 from asparagine to lysine causes the failure of some CPV antibodies [27]; therefore, this mutation may cause vaccine failure in the recent CPV-2 strains. Nowadays, NS1 (I60V, Y544F, E545V, L630P) and VP2 (A5G, F267Y, N297A, Y324I, and Q370R) are some of the important mutations, raising concerns in Asia and Europe [28].

Viral genome always goes through different mutations, which may help them evade the immune system memory. According to several studies, the existing vaccine against CPV-2 does not have absolute efficacy [29]. A potential vaccine failure could be caused by amino acid mutations in protein’s active sites, such as loops. N93K is also located at the active site of protein, near loop 2. Therefore, it has the potential to be a cause of vaccine failure. In conclusion, this study investigates the whole genome sequences of CPV-2 in order to discuss the observed mutations in NS1, VP1, and VP2, and their potential contribution to vaccine failure. However, more pathological research needs to be carried out to confirm the roles assumed for these amino acid substitutions.

## Supplementary Information

**Additional file 1**. List of all isolation with accession number.

## Data Availability

The data sets used and analyzed during the current study are available from the corresponding author on reasonable request.
